# A new cellular model to follow Friedreich's ataxia development in a time-resolved way

**DOI:** 10.1242/dmm.020545

**Published:** 2015-07-01

**Authors:** Tommaso Vannocci, Nathalie Faggianelli, Silvia Zaccagnino, Ilaria della Rosa, Salvatore Adinolfi, Annalisa Pastore

**Affiliations:** 1 Molecular Structure Division, MRC National Institute for Medical Research, The Ridgeway, London NW7 1AA, UK; 2 Department of Basic and Clinical Neurosciences, Kings College London, London NW7 1AA, UK

**Keywords:** Cellular model, Disease development, Frataxin, Genetic models, Zinc fingers

## Abstract

Friedreich's ataxia (FRDA) is a recessive autosomal ataxia caused by reduced levels of frataxin (FXN), an essential mitochondrial protein that is highly conserved from bacteria to primates. The exact role of frataxin and its primary function remain unclear although this information would be very valuable to design a therapeutic approach for FRDA. A main difficulty encountered so far has been that of establishing a clear temporal relationship between the different observations that could allow a distinction between causes and secondary effects, and provide a clear link between aging and disease development. To approach this problem, we developed a cellular model in which we can switch off/on in a time-controlled way the frataxin gene partially mimicking what happens in the disease. We exploited the TALEN and CRISPR methodologies to engineer a cell line where the presence of an exogenous, inducible *FXN* gene rescues the cells from the knockout of the two endogenous *FXN* genes. This system allows the possibility of testing the progression of disease and is a valuable tool for following the phenotype with different newly acquired markers.

## Abstract (precis)

**Summary:** This paper describes the development of a new cellular model to study the neurodegenerative Friedreich's ataxia based on the use and development of new CRISPR and TALEN platforms.

## INTRODUCTION

Friedreich's ataxia (FRDA) is an autosomal recessive neurodegenerative disease with an occurrence of 1 in 50,000 individuals and onset usually before 25 years of age (Cossée et al., 1997). The pathophysiology of the disease is associated with progressive neurodegeneration, spinocerebellar ataxia, sensory loss and hypertrophic cardiomyopathy (Pandolfo, 2009).

A homozygous GAA triplet expansion in the first intron of the frataxin gene (*FXN*) is the cause of the disease in 96% of individuals with FRDA. This expansion is probably responsible for epigenetic modifications upstream of the expansion and consequent silencing of the *FXN* gene (Saveliev et al., 2003; Sandi et al., 2013). Normal individuals carry around 40 copies of the GAA triplet but individuals with FRDA present larger expansions, usually up to 600-900 GAA repeats (Campuzano et al., 1996). The size of the expansion is directly correlated to the speed of disease progression, indicating a connection between frataxin expression levels and severity (Filla et al., 1996). A small fraction (∼3%) of individuals with FRDA is heterozygous with one allele carrying the triplet expansion and the other a loss-of-function mutation. Interestingly, lack of FRDA patients with homozygous nonsense mutations and embryonic lethality observed in *Fxn*-knockout mice indicate the need for an at least partial expression of the *FXN* gene at the early stages of embryonic development (Cossée et al., 1999, 2000).

The tissues that are mostly affected by FRDA are sensory neurons and cardiomyocytes, which are both tissues with high content of mitochondria and high metabolism (Perdomini et al., 2013). At the cellular level, the disease is characterized by mitochondrial iron accumulation, increased oxidative stress and abnormalities in iron-sulphur (Fe-S) cluster biogenesis (Pandolfo and Pastore, 2009; Martelli and Puccio, 2014). Despite extensive studies, it is still unclear which of these three cellular dysfunctions are the main promoters of FRDA and which are secondary phenotypes (Pandolfo and Pastore, 2009; Vaubel and Isaya, 2013; Martelli and Puccio, 2014). Complete understanding of the disease is also complicated by the fact that the exact role and primary function of FXN remains unclear, although the protein is known to have iron-binding properties and to play an important role in the regulation of Fe-S cluster biogenesis (Pastore and Puccio, 2013). FXN is an essential protein highly conserved from bacteria to primates. It is nuclearly encoded and produced in the cytoplasm as a 210 amino acid protein that is then imported into the mitochondrion where it is matured into its final form (residues 81-210) by the mitochondrial processing peptidase (MPP) (Schmucker et al., 2008).

Many different approaches have been developed in the attempt to identify the function of frataxin and its role in the onset of FRDA. They were based either on biophysical and structural studies (Pastore and Puccio, 2013) or on animal and cellular models (Perdomini et al., 2013). *In vivo* iPSCs derived from FRDA patients (Ku et al., 2010; Hick et al., 2013) and multiple conditional knockout mice (Pook et al., 2001; Puccio et al., 2001; Simon et al., 2004; Al-Mahdawi et al., 2008) were extensively used to investigate the effects of frataxin depletion. However, due to the intrinsic characteristics of these models, most studies could not explore the time-course of the disease and the phenotype was tested at few temporally spaced time points (Perdomini et al., 2013). Therefore, the early events that lead to the disease onset have yet to be fully understood. This knowledge would be crucial to allow us to discriminate between the causes and the effects of the disease (Pastore and Adinolfi, 2014).

Here, we describe a new cellular model in which expression of frataxin can be regulated at will by engineering a cell line where the presence of an exogenous, inducible *FXN* gene integrated in the genome is used to compensate for the bi-allelic knockout of the endogenous *FXN* gene. The knockout was carried out by gene editing using a targeting construct with a selectable marker and sequential rounds of selection. Two different custom gene-editing platforms were used to boost targeting frequencies and thus facilitate the development of the cell line model to overcome the low frequencies of knockout induced by traditional gene targeting techniques: the transcription activator-like effector nucleases (TALENs) (Cermak et al., 2011) and the clustered regularly interspaced short palindromic repeats/Cas9 (CRISPR/Cas9) (Mali et al., 2013). They are the latest two members of the growing family of genome editing systems (Gaj et al., 2013).

**TRANSLATIONAL IMPACT**

**Clinical issue**

Friedreich's ataxia (FRDA) is a recessive autosomal form of ataxia that is characterized by neurodegeneration, loss of muscle coordination and hypertrophic cardiomyopathy. It is caused by reduced levels of frataxin (FXN), an essential mitochondrial protein that is involved in the regulation of iron-sulfur cluster biogenesis and that is highly conserved from bacteria to primates. The exact role of FXN and its primary cellular functions in sensory neurons and cardiomyocytes – the cell types that are mostly affected in FRDA – remain largely unclear. However, this information is crucial for designing therapeutic approaches for FRDA and for understanding the molecular mechanisms of this disease. One main difficulty is that current experimental models are not able to follow the progression of the disease, which could allow researchers to distinguish causative mechanisms from secondary effects, thus gaining more insights into the molecular basis of FRDA.

**Results**

This work describes the development of a new cellular model of FRDA to study the disease progression and phenotypes in a time-controlled manner. The authors used a human cell line in which they first introduced an exogenous inducible form of the gene and then knocked down the endogenous *FXN* gene by using two gene-editing platforms: the transcription activator-like effector nucleases (TALENs) and the clustered regularly interspaced short palindromic repeats/Cas9 (CRISPR/Cas9). Immunofluorescence and western blot analyses revealed that this approach successfully enables switching off and on the *FXN* gene in the cells in a time-controlled way, partially mimicking what happens in the disease.

**Implications and future directions**

This new cellular model of FRDA offers the possibility of studying the effects of FXN depletion at different time points to better understand the early stages and progression of the disease. It has also the potential to use available markers, e.g. indicators of iron-sulphur cluster biogenesis, to characterize and follow FRDA phenotypes over time. In addition, the genome-editing techniques used here could be adapted for use in induced pluripotent stem cells, which can be differentiated in either neuronal or cardiac cells, allowing for the time-resolved characterization of FRDA phenotypes in relevant cell types. 

Although very different from one another, they share the same basic molecular mechanism. Both are characterized by a DNA recognition domain that faithfully binds the desired target sequence and a cleaving domain that produces the double-strand break (DSB) (Cermak et al., 2011). They create DSBs at specific genomic loci, promoting homologous recombination, and efficiently increase the frequencies of induced genome modifications (Gaj et al., 2013). The DNA-binding domain of TALENs is directly derived from the TAL effector proteins secreted by Xanthomonas bacteria, a class of plant pathogens. It is made of highly conserved protein ‘modules’ of 33-34 amino acids with just two repeat variable di-residues (RVDs, positions 12th and 13th) that allow for recognition of a single nucleotide (Boch et al., 2009). The modularity of TAL effectors can be implemented to generate DNA-binding domains of around 20 different units that confer a high degree of DNA-binding specificity. TAL domains can be combined with an unspecific DNA-cleavage domain (*Fok*I) to produce DSBs at the desired genomic locus (Cermak et al., 2011).

The CRISPR/Cas9 platform is directly based on a prokaryotic immune system common to many bacteria and archea that protects the organism from exogenous genetic elements. In this case, the DNA specificity is granted by RNA guides of around 20 nucleotides homologous to the target DNA to be cleaved that guide the unspecific nuclease Cas9 towards its target (Horvath and Barrangou, 2010).

Using these tools, we obtained what may be a powerful tool to follow the progression of FRDA, which may finally allow us to understand the molecular basis of this disease. The model will allow us to quantify the effects of reduced levels of frataxin in a time-controlled way.

## RESULTS

### Development of an inducible frataxin gene and stable transfection

Complete silencing of the endogenous *Fxn* gene induces interruption of cell division and subsequent cell death in cell models (Calmels et al., 2009), and is a lethal mutation that leads to premature death in mouse embryos (Cossée et al., 2000). The first step towards the development of our cell model was thus to introduce an inducible exogenous *FXN* gene that would rescue cells that underwent homozygous knockout of the endogenous *FXN* gene from premature cell death. We decided to rely on the ‘Flp-in T-REx’ (HEK293) system (Life Technologies) to achieve a tightly controlled expression of exogenous *FXN* as it allows generation of stable mammalian cell lines using Flp-recombinase-mediated integration of an expression cassette at a specific genomic location. This system has the advantage of creating isogenic stable cell lines in which controlled, high levels of expression of the desired gene are obtained.

The exogenous *FXN* cassette to be used in the inducible system was created using the cDNA of human full-length FXN (amino acids 1-210, RefSeq FXNNM_000144, hereafter called cFXN). The cDNA was cloned into the pcDNA5/FRT/TO plasmid and was co-transfected with the Flp-recombinase expression vector (pOG44) in Flp-in T-REx cells. The pcDNA5/FRT/TO plasmid harbours a hygromycin cassette that is expressed only when the plasmid is correctly integrated in the FRT locus carried by the Flp-in T-REx cells. Cells that underwent successful integration were selected for 15 days in 10 cm plates using hygromycin. Single colonies were isolated and expanded. The cell line generated was dubbed T-REx293-cFXN.

### Characterization of the inducible FXN cell line

**Fig. 1.**
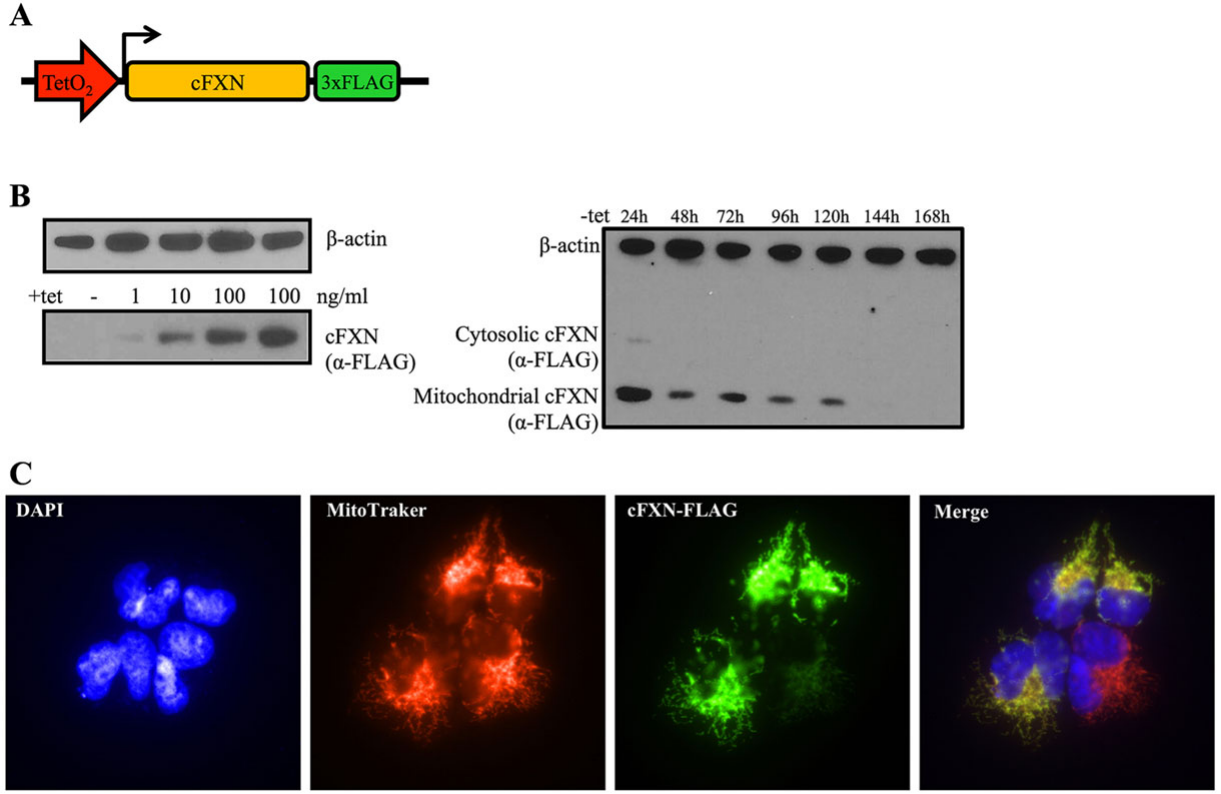
**Testing controlled exogenous FXN expression.** (A) The exogenous inducible cFXN cassette. The inducible promoter TetO_2_ is shown in red, the cDNA frataxin gene in yellow and the 3× FLAG tag in green. (B) Western blot showing the correlation between tetracycline concentrations and levels of cFXN expression (left). Stability of the exogenous frataxin protein after suspension of tetracycline induction (right). Antibodies against β-actin were used as controls. (C) Immunofluorescence confirms the mitochondrial localization of the induced frataxin protein. The nuclear DNA was stained with DAPI (blue), mitochondria with MitoTracker red (red) and cFXN-FLAG protein with monoclonal anti-FLAG (primary antibody) and AlexaFluor-488 (secondary antibody) (green).

The cFXN cassette is under the control of the tetracycline-regulated promoter CMV-TetO2 (Yao et al., 1998) that is bound and repressed, in absence of tetracycline, by the tet-repressor protein expressed by the stably integrated regulatory plasmid pcDNA6/TR. A 3× FLAG tag (peptide sequence DYKDDDDK) was cloned downstream of the gene (i.e. at the C terminus of the expressed frataxin protein) to monitor the levels of expression of the cFXN cassette over time (Fig. 1A). We confirmed expression of the cFXN cassette under induction with increasing amounts of tetracycline by detection of the FLAG tag in western blot assays using a specific anti-FLAG antibody. The resulting expression of cFXN was dependent on the concentration of tetracycline, indicating a good level of control on the induction of the exogenous gene and therefore giving us the ability to modulate the amount of expressed protein. Levels of protein expression were assessed by comparing them with those of the endogenous β-actin protein (Fig. 1B). Subsequently, we assessed the half-life of the induced FXN protein in cells after removal of tetracycline from the culture medium. T-REx293-cFXN cells were induced for 48 h with tetracycline. Cell samples were then collected every 24 h (Fig. 1B). Already after 24 h the unprocessed, cytosolic form of FXN (amino acids 1-210) was barely detectable, while the mitochondrial form (amino acids 81-210) persisted for a period of 5 days before becoming undetectable. Finally, we confirmed mitochondrial localization of the matured exogenous FXN protein by immunofluorescence (Fig. 1C).

### Design of FXN-specific TALENs and CRISPRs

**Fig. 2.**
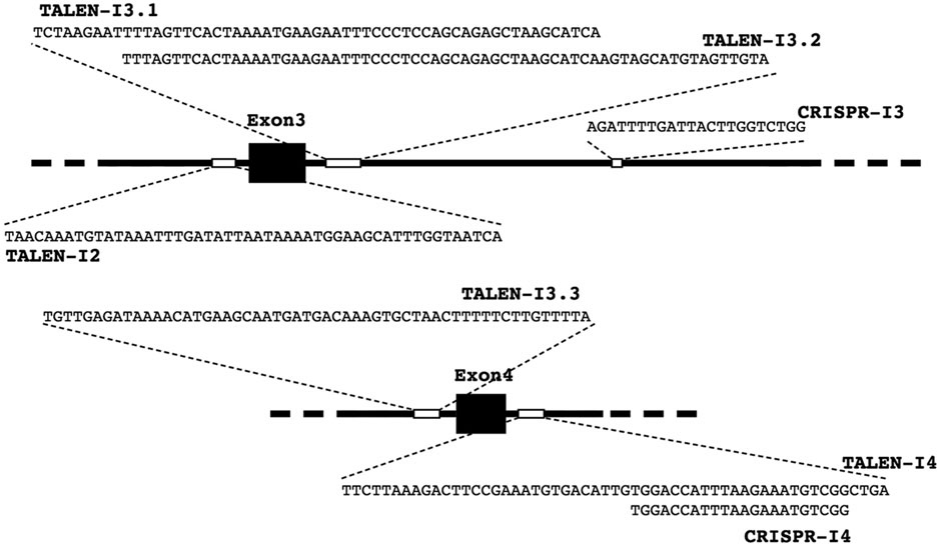
***FXN* target sequences.** Exons 3 and 4 of *FXN* with surrounding areas drawn to scale. Exons are shown as black boxes; introns are black lines. The target sequences for both TALENs and CRISPRs are graphically represented as small white boxes and also as full nucleotide sequences.

Identification of suitable target sequences to be used with the TALEN platform was carried out by querying the web-based software TALE-NT 2.0 (Doyle et al., 2012) with the human *FXN* gene (Gene ID: 2395). The software identifies possible sites using parameters based on a previously described protocol (Cermak et al., 2011): the length of the target sequences for each TAL-binding domain (half binding domains, either on the right or left of the cutting site) was set to be between 15 and 20 bp. The spacer sequence, the nucleotide region where the cutting will occur, was set to a length of 10-16 bp. TALENs predicted by TALE-NT 2.0 using these settings would identify target sequences of at least 40 bp, a nucleotide length that guarantees high specificity and reduces the risk of off-target cleavage events (Fig. 2).

Of all possible target sequences originated by TALENT 2.0, five were chosen for their relative positions to FXN exons and, when possible, for the presence of suitable unique restriction sites for the following cutting efficiency assay (see below). The relative TAL-binding domains were generated by the Golden Gate approach using the TAL archive provided by Addgene. Four out of the five pairs of TAL-binding domains (one left and one right for each pair) were cloned in the expression vector pcGoldyTALEN (TALEN-I3.1, -I3.2, -I3.3 and -I4) (Bedell et al., 2012; Carlson et al., 2012), whereas TALEN-I2 was created using the pCS2TAL3-RRR and -DDD expression vectors (Dahlem et al., 2012). These last two expression vectors encode two mutated half nuclease domains that transform the homodimer *Fok*I into an obligate heterodimer. These alternative expression plasmids were chosen after preliminary results on the cutting efficiencies of the first four TALENs showed unsatisfactory levels of target sequence cleavage.

An alternative engineered endonuclease platform, CRISPR/Cas9, was used to increase the chances of effectively targeting the *FXN* gene. Two different CRISPRs (CRISPR-I3 and -I4) were generated using a kit provided by Addgene according to the Church lab's approach (Fig. 2) (Mali et al., 2013). The target sequences were selected from a library of more than 190,000 sequences previously created to facilitate the selection process because of their proximity to the FXN exons (Mali et al., 2013).

### Detection of TALEN- and CRISPR-induced FXN-specific modifications

**Fig. 3.**
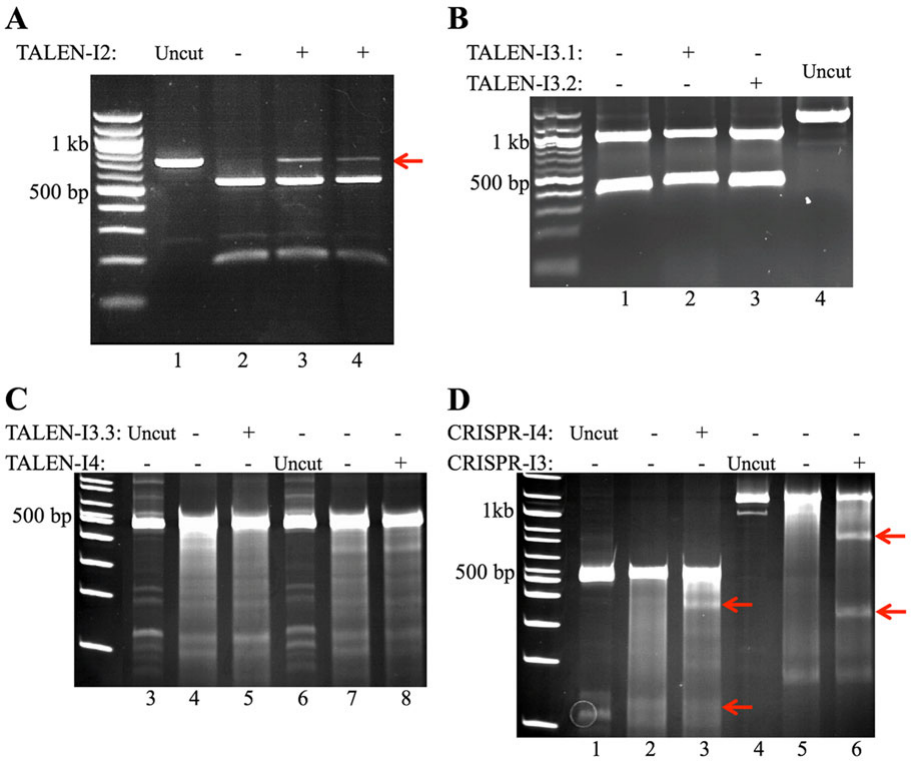
**TALEN and CRISPR cutting efficiencies.** 2×10^5^ T-REx293-cFXN cells were plated in six-well plates 48 h before transfection. Cells were transfected with 2.5 μg of DNA. Genomic DNA isolated from cells 48 h after transfection was used as a template to amplify DNA fragments surrounding the relative TALENs and CRISPRs target sequences by PCR. (A) PCR products for the region surrounding TALEN-I2 target sequence were digested with the unique restriction enzyme *Ase*I. Lane 1, undigested PCR product used as a reference; lane 2, digested PCR product from negative control; lanes 3 and 4, digested PCR products from two separate cells samples transfected with TALEN-I2 expression vectors. (B) PCR products for the region surrounding TALEN-I3.1 and TALEN-I3.2 target sequences were digested with the unique restriction enzymes *Xmn*I and *Bpl*I, respectively. Lane 1, undigested PCR product used as a reference; lane 2, *Xmn*I-digested PCR product from negative control; lane 3, *Xmn*I-digested PCR product from cells transfected with TALEN-I3.1; lane 4, *Bpl*I-digested PCR product from cells transfected with TALEN-I3.2. (C) PCR products from genomic DNA of TALEN-I3.3 and -I4 transfected cells were digested with Surveyor endonuclease. Lanes 3 and 6, undigested PCR products; lanes 4 and 7, Surveyor-digested PCR products from negative controls; lanes 5 and 8, Surveyor-digested PCR products from cells transfected with TALEN-I3.3 and -I4 vectors, respectively. Lanes 1 and 2 have been excised for clarity. (D) PCR products from genomic DNA of CRISPR-I4- and -I3-transfected cells were digested with Surveyor endonuclease. Lanes 1 and 4, undigested PCR products; lanes 2 and 5, Surveyor-digested PCR products from negative controls; lanes 3 and 6, Surveyor-digested PCR products from cells transfected with CRISPR-I4 and -I3 vectors, respectively. Red arrows indicate the presence of TALEN-I2-, CRISPR-I3- and CRISPR-I4-induced indels in the relative amplicons.

To assess cleavage of *FXN* by either TALENs or CRISPRs in human cells, we transfected T-REx293-cFXN cells with either TALEN or CRISPR expression vectors. Transfected cells were collected after 48 h and genomic regions surrounding TALEN and CRISPR target sequences of *FXN* were amplified by PCR. Two different approaches were adopted to detect the formation of indels resulting from inaccurate NHEJ at the target site. When possible, NHEJ-induced loss of unique restriction sites at the target sequences was detected by restriction digestion of the amplicons (TALEN-I2, -I3.1 and -I3.2) followed by gel electrophoresis. The presence of indels was confirmed by identification of uncut product (Fig. 3A,B).

Alternatively, we used the Surveyor nuclease assay (Trangenomic) to detect the formation of indels (TALEN-I3.3, -I4, CRISPR-I3 and -I4). Surveyor nuclease, which cleaves short regions of heteroduplex DNA, cleaved the PCR amplicons into two fragments of sizes expected for heteroduplex formation at the target sites of the endonucleases (Fig. 3C,D).

Cutting efficiencies varied greatly for each endonuclease tested. TALEN-I3.1, -I3.2, -I3.3 and -I4 completely failed to show any sign of cleavage activity (Fig. 3B,C). Digested PCR products originating from genomic DNA of TALEN-I2-treated cells instead showed the presence of a clear uncut band (Fig. 3A). The negative control, a sample obtained by transfecting cells with pmaxGFP vector, showed complete digestion of the amplicon, indicating that only TALEN-I2 related amplicons carried indels. Interestingly, TALEN-I2 was the only TALEN developed using the alternative expression vectors pCS2TAL3-RRR/DDD, indicating a possible problem with the pcGoldy-TALEN vector.

The Surveyor assay performed on amplicons derived from CRISPR-treated cells indicated that both CRISPR-I3 and -I4 successfully cut their relative target sequences (Fig. 3D). Surveyor-induced cleavage of amplicons was detected only in samples derived from cells transfected with the CRISPR expression vectors and not in the relative negative controls. Although it is not possible to quantitatively compare the results obtained from the TALEN-I2, CRISPR-I3 and -I4 due to the differences between the performed assays (loss of restriction site and Surveyor), CRISPR-I3 seemed to show the greatest cutting efficiency.

### Homology directed knockout of the endogenous *FXN* gene

**Fig. 4.**
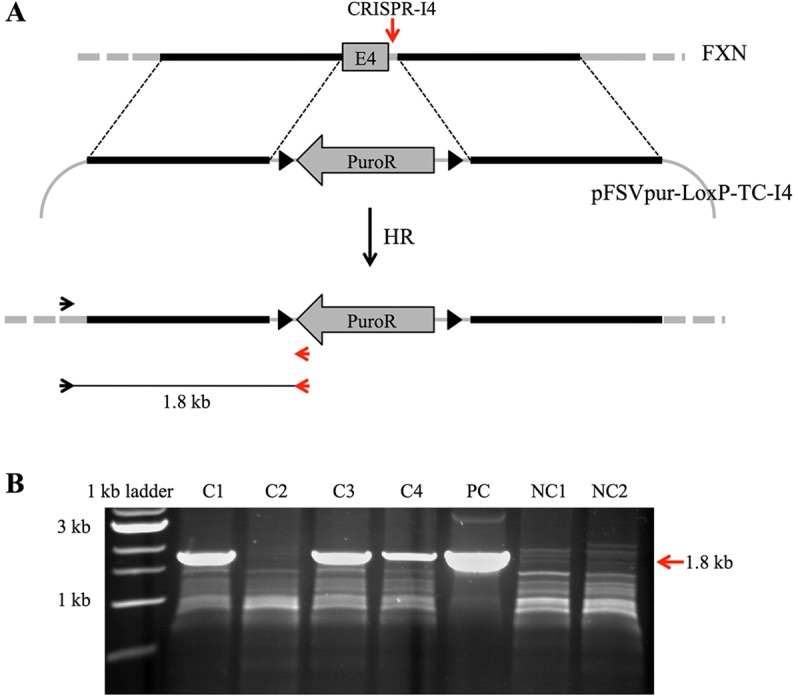
**CRISPR-I4 promotes targeted disruption of the *FXN* gene.** (A) Exon 4 of FXN is depicted (top) with flanking regions in black (homologous to pFSVpur-LoxP-TC-I4) or grey. The vertical red arrow shows the CRISPR-I4 target site. The targeting construct (pFSVpur-LoxP-TC-I4) is shown below the *FXN* gene with dotted lines indicating regions of homology with the target locus. LoxP sites flanking the puromycin resistance are represented as two black triangles. The result of CRISPR-I4-promoted homologous recombination between pFSVpur-LoxP-TC-I4 and the target locus is shown at the bottom. Black and red horizontal arrows indicate target-specific and construct-specific PCR primers, respectively. Underneath are the expected PCR product and its expected size (1.8 kb). (B) Agarose gel of the PCR screening assay for detection of targeted events. C1, C3 and C4 products show the expected 1.8 kb band amplified from CRISPR-I4-, Cas9- and pFSVpur-LoxP-TC-I4-transfected cells, respectively. The 1.8 kb PCR product indicative of a successful targeting is indicated by the red arrow. C2 shows an example of untargeted event. PC shows the PCR product obtained using as a template the positive control plasmid (pFSVpur-TC-I4-PC). NC1 and NC2 (negative controls) show the absence of the characteristic 1.8 kb PCR band. These last two samples were obtained from cells transfected with only Cas9 and pFSVpur-LoxP-TC-I4 vectors.

We next proceeded to produce gene targeting at the endogenous *FXN* gene using the most suitable endonuclease of the successful TALEN-I2, CRISPR-I3 and -I4. CRISPR-I4 was chosen because of the position of its target sequence being relatively close to *FXN* exon 4 and its cutting efficiency. To achieve successful knockout of the target gene, we made a targeting construct (pFSVpur-LoxP-TC-I4) designed to introduce a puromycin resistance (Puro^R^) cassette at the CRISPR-I4 target site (Fig. 4A). The targeting construct was designed to carry the Puro^R^ cassette flanked by two arms of homology specific for the targeted region of *FXN*. Additionally, two LoxP sites were cloned immediately upstream and downstream of the selectable marker. Expression of Cre recombinase in transfected cells will allow removal of the Puro^R^ cassette. The pFSVpur-LoxP-TC-I4 construct was designed to completely remove, in case of successful CRISPR-I4-mediated targeting, the endogenous FXN exon 4 and replace it with the Puro^R^ cassette.

We co-transfected T-REx293-cFXN cells with 2.5 μg of DNA, varying the ratio of CRISPR-I4/hCas9 vectors and targeting construct (Table 1). Negative controls were performed by transfecting cells with the targeting construct and the hCas9 vector only. After transfection, cells were plated on 10 cm plates and selected in puromycin for 15 days. Single colonies (20 for each experiment) were collected and expanded to test possible targeted events by a specific PCR assay (Fig. 4B; Table 1). The PCR assay was carried out using two primers: one target specific and one construct specific. PCR products of the correct size (1.8 kb), therefore, would be produced only in the case of successful targeting and not for randomly integrated events. Positive controls for the PCR screening were obtained by mixing, in the PCR reaction, the pFSVpur-TC-I4-PC plasmid with genomic DNA originated from untransfected cells. Ten and nine clones out of 20 for experiments 1 and 2, respectively, gave positive results in the PCR assay, while none of the negative controls produced PCR products of the expected size. Although the number of screened clones was not statistically significant, the targeting efficiency, overall, was ∼50%. Moreover, the targeting frequencies seemed not to be affected by the ratio of transfected CRISPR-I4/pFSVpur-LoxP-TC-I4 vectors used in the two different experiments (Table 1).

**Table 1.**
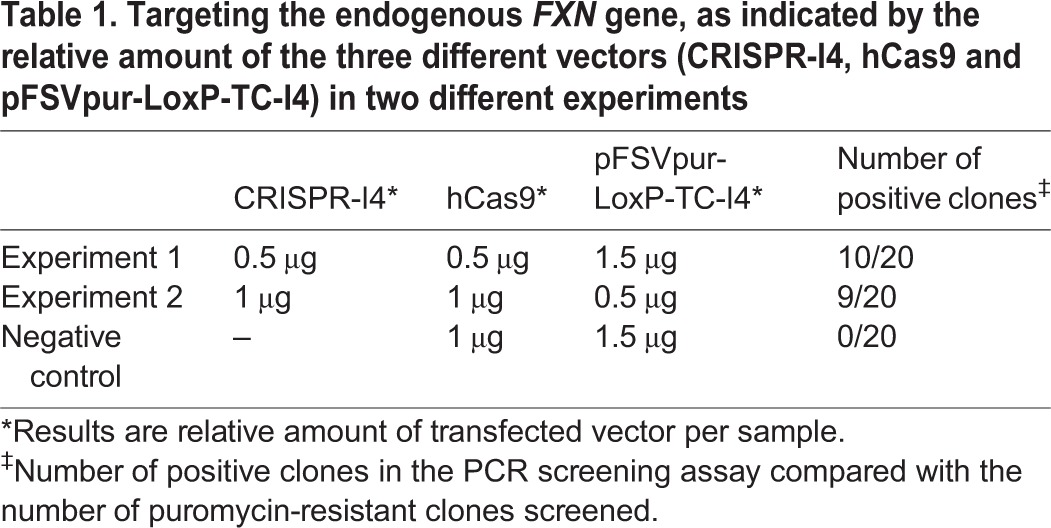
**Targeting the endogenous *FXN* gene, as indicated by the relative amount of the three different vectors (CRISPR-I4, hCas9 and pFSVpur-LoxP-TC-I4) in two different experiments**

## DISCUSSION

Since its beginning more than 30 years ago (Thomas et al., 1986; Thomas and Capecchi, 1987), genetic engineering has become an invaluable tool in many different scientific and industrial fields. Developments in genetic manipulation techniques have also pushed the boundaries of what is possible by gene engineering. These include a vast number of applications from the study of gene function and the development of genetically modified organisms, to gene therapy approaches such as gene addition and gene correction (Yanez and Porter, 1998; Hacein-Bey-Abina et al., 2010; Nemudryi et al., 2014). Probably the latest and more revolutionary advancement in this field was the creation of a new family of molecular tools that allows precise editing of virtually any desired genomic sequence (Gaj et al., 2013; Nemudryi et al., 2014), starting from the seminal 2006 paper on zinc finger nucleases (ZFNs) (Wright et al., 2006) to the most recent introduction of CRISPRs (Mali et al., 2013). These ‘molecular scissors’ have allowed a cheaper, faster and more accurate alternative to traditional gene targeting based on simple homologous recombination. The use of TALEN/CRISPR nucleases is also a valuable alternative to RNAi (RNA interference), especially for kinetic studies where the phenotype has to be followed over a long time and where uncontrolled levels of residual protein expression could affect the outcome of the performed analysis. Although a valuable approach for the study of gene function by knockdown, traditional RNAi is in fact unsuitable in long-term studies because of its inability to produce tightly controlled, long-term inhibition of the target gene and by providing only incomplete repression of protein expression (Gaj et al., 2013).

In this study, we aimed to develop and characterize FXN-specific endonucleases for the generation of stable knockout cell lines to be used in functional studies. We were prompted by the necessity of distinguishing between causative processes and secondary effects in the development of FRDA (Pastore and Adinolfi, 2014). This necessity is particularly crucial with this disease because, almost 20 years after identification of the *FXN* gene, we still ignore the disease mechanism or, more dramatically, what actually kills people other than the reduced expression of FXN. Independent cellular and animal models all revealed that FXN deficiency primarily leads to defects in iron-sulphur cluster-containing proteins, but gave contradictory results on whether accumulation of iron deposits is a primary or secondary effect, and on the role of oxygen free-radicals (ROS). In yeast, a defect in respiratory efficiency was detected that follows disruption of iron-sulphur cluster maturation (Muhlenhoff et al., 2002), whereas iron-sulphur cluster deficit was described to precede the first evidence of a cardiac dysfunction in mouse (Martelli et al., 2012). Interestingly, no evidence of oxidative damage was observed in mouse and *Drosophila* models (Anderson et al., 2005; Llorens et al., 2007), which fails to clarify whether ROS is essential or even important to trigger the FRDA phenotype. This conflicting evidence demanded the possibility of capturing the early stages of disease and following disease development in a time-resolved way starting from a precise time point. Previous attempts to achieve this goal involved the use of conditional knockouts developed by several different groups (Puccio et al., 2001; Muhlenhoff et al., 2002; Martelli et al., 2012) and an RNAi-mediated suppression strategy in *Drosophila* (Anderson et al., 2005; Llorens et al., 2007). Our system, however, presents undoubtable advantages over these models because of the complete efficiency of gene silencing and its flexibility.

We used a parallel approach to increase the chance of obtaining an effective knockout of the target gene and compare the efficiency of TALEN and CRISPR platforms. In our hands, although both CRIPSR-I4 and TALEN-I4 targeted the same region, downstream of the exon 4, they showed distinctively different cleavage efficiency. This cannot be ascribed to intrinsic characteristics of the different targeted loci (accessibility and epigenetic modifications), indicating a distinctive higher efficiency of CRIPSR-I4 at inducing DSBs. Differences were also identified in the use of different TALEN expression vectors. While TALENs developed using the pcGoldy-TALEN vector (TALEN-I3.1, -I3.2, -I3.3 and -I4) performed poorly, TALEN-I2, produced using the pCS2TAL3-RRR/DDD vector, showed appreciable cutting activity. Both types of expression vector harbour a modified TALEN backbone with truncated N- and C-termini flanking the RVD cloning site, which has enhanced binding activity (Miller et al., 2011; Mussolino et al., 2011) compared to the traditional backbone used by Cermak et al. (2011). Additionally, pCS2TAL3-RRR/DDD vectors carry a modified nuclease domain that renders the homodimeric *Fok*I an obligate heterodimer. This modification confers higher cleavage activity and, at the same time, reduces the risk of off-target cleavage that is known to increase the risk of undesired mutations and cell death (Doyon et al., 2011).

Of the three newly developed endonucleases that successfully induced significant FXN-targeted modifications (TALEN-I2, CRISPR-I3 and CRISPR-I4), we chose CRISPR-I4 for the proximity of its target sequence to FXN exon 4. This allowed us to create a targeting construct (pFSVpur-LoxP-TC-I4) that, when integrated by homologous recombination, excised the exon completely, replacing it with a puromycin resistance cassette. Although integration of the selectable marker per se was enough to disrupt the *FXN* gene, the need to produce knockout of both FXN alleles still required two rounds of transfection with CRISPR-I4 and the targeting construct because simultaneous homozygous FXN knockout is a rare event. The presence of the puromycin cassette flanked by two Lox-P sites allowed us to select the targeted cells in the first round followed by Cre recombinase-mediated excision of the puromycin cassette and a second round of targeting using the same pFSVpur-LoxP-TC-I4 construct. The targeting experiments carried out with CRISPR-I4 and pFSVpur-LoxP-TC-I4 showed a targeting frequency of ∼50% to be compared to a 0% frequency when cells were transfected with only pFSVpur-LoxP-TC-I4 targeting construct. This study, therefore, proves the feasibility of successfully performing gene editing at the *FXN* locus.

The HEK293 cell line used in this project has some limitations as an accurate representation of neuronal and cardiac cells, the two main tissues affected in individuals with FRDA. It was nonetheless selected because of its ease to work with, which allowed us to fully assess the gene engineering techniques and initially test our perspectives to assess disease progression. The techniques developed here are incredibly flexible and will be used in the near future to generate novel models based on cell types more relevant to the study of FRDA. Our objective is to adapt the system to either stem cells or induced pluripotent stem cells (iPSCs) in order to develop a cell model that can be later on differentiated in either neuronal or cardiac cells, allowing for the time-resolved characterization of FRDA in relevant tissues and their comparison.

We are thus now in a strong position to follow and understand the early stages in the development of FRDA. We also plan to monitor the effects of FXN depletion using different biomarkers chosen amongst the classical and newly developed ones. Along with the established aconitase assay (an indicator of iron-sulphur cluster biogenesis), we can, for example, test the new family of markers developed for ROS detection, which were already shown to be successful in FRDA studies (Albrecht et al., 2014). We are thus confident that our work will provide an important contribution to the study and the diagnosis of FRDA.

## MATERIALS AND METHODS

### DNA constructs development

The pcDNA5-cFXN-FLAG expression vector was created by amplifying the full-length *FXN* cDNA gene carried by the pTLXI plasmid [kindly donated by Dr Mark Pook (Brunel University, London), FXN RefSeq FXNNM_000144) using Q5 Hot Start High-Fidelity DNA polymerase (NEB) with primers 5′-ATATTGGATCCGCCACCATGTGGACTCTCGGGCGC-3′ and 5′-ATATTCTCGAGGGCGGCAGCATCTTTTCCGGAATAGGCC-3′, digested with *Bam*HI and *Xho*I (NEB) and cloned in a modified pcDNA5™/FRT/TO inducible expression vector that carries at the C terminus of the gene of interest a 3× FLAG tag.

To make the pFSVpur-LoxP-TC-I4 targeting construct, Q5 Hot Start High-Fidelity DNA polymerase (NEB) was used to PCR amplify the two homology arms from genomic DNA extracted from Flp-InT-REx-293 cells. The left homology arm (1.6 kb) was amplified with primers 5′-ATATTGAGCTCCATGATCCTGCCACTGCTGC-3′ and 5′-ATATTGCGGCCGCGGAGTTTGTGTAGGAAGAGCTTTGC-3′, digested with *Sac*I and *Not*I restriction enzymes (NEB) and cloned into pFSVpur-LoxP vector (kindly donated by Dr Andrew Porter's lab, Imperial College London) to make pFSVpur-LoxP-TC-I4-left. The right homology arm (1.6 kb) was amplified with primers 5′-ATATTCTCGAGCTGACACCTGTAATCCCAACAC-3′ and 5′-ATATTGGTACCGGGCAGTGTGTCCAAATACGATATG-3′, digested with *Xho*I and *Kpn*I (Neb), and cloned into pFSVpur-LoxP-TC-I4-left to create the final targeting construct pFSVpur-LoxP-TC-I4. The pFSVpur-TC-I4-PC construct to be used as a positive control during the PCR screening assay was created by PCR amplification with Q5 Hot Start High-Fidelity DNA polymerase (NEB) using primers 5′-CTCTCTGGAGGGCTAGCTGTAAAACGACGGCCAGT-3′ and 5′-CTGGGACTACACTGGCGTTACCCAACTTAATC-3′ followed by digestion with *Dpn*I (NEB) and blunt ligation with T4 Ligase (NEB).

### Cell culture and transfection

Flp-InT-REx-293 cells (Life Technologies) were cultured in DMEM supplemented with 10% foetal bovine serum (FBS, Gibco), 10 mM sodium pyruvate (Gibco), 20 mM L-glutamine (Life Technologies), 400 IU/ml penicillin-streptomycin (Life Technologies), 10 ml of a 100× MEM-NEAA solution (Gibco), 15 μg/ml blasticidin (Life Technologies) and 100 μg/ml zeocin (Life Technologies).

Stable and transient transfections of cells were carried out using Lipofectamine 2000 (Life Technologies) following the manufacturer's instruction. Briefly, 2×10^5^ cells were plated in a 6-well plate and transfected 48 h afterwards with 2.5 μg of total DNA. Transfection efficiencies were estimated by transfecting 2.5 μg of a GFP expression vector (pmaxGFP, Lonza) and flow cytometric analysis 48 h after transfection. Efficiency ranged between 60% and 80%.

### Generation of the stable T-REx293-cFXN cell line

Flp-InT-REx-293 cells were transfected using Lipofectamine, as previously described, with 2.2 μg of pOG44 vector (expressing the Flp recombinase, Life Technologies) and 0.3 μg of pcDNA5-cFXN-FLAG expression vector. The vector harbour a FRT site that is used to stably integrate it by Flp-mediated recombination with a specific FRT site already present in the genome of the Flp-InT-REx-293 cells. Selection of suitable clones was carried out for 12 days in 10 cm plates containing DMEM supplemented with 100 μg/ml hygromycin (Life Technologies) and 15 μg/ml blasticidin (Life Technologies).

### Western blots

Assessment of cFXN expression in isolated T-REx293-cFXN clones was carried out by plating of 1.5×10^5^ cells in 12-well plate in presence of 100 μg/ml hygromycin and 15 μg/ml blasticidin, and increasing amounts (1 to 100 ng/ml) of tetracycline (Sigma-Aldrich). Cells were collected after 48 h, pelleted by centrifugation and resuspended in 75 µl of RISC buffer: 20 mM Tris-HCl (pH 7.5), 150 mM NaCl, 0.5% NP-40, 2 mM MgCl_2_, 1 mM DTT, 1 mM PMSF (all reagents were purchased from Sigma-Aldrich) and EDTA-free protease inhibitor (Roche). Cells were lysed for 1 h at 4°C with shaking. Samples were centrifuged for 10 min at full speed to remove pellet and protein concentration in the supernatant was measured using Bio-Rad protein assay (OD_595 nm_). Total protein (25 and 50 μg) was subjected to SDS-PAGE using precast 12% polyacrylamide gels (Life Technologies). Samples were then transferred on nitrocellulose membrane (0.2 μm pore size, Life Technologies) for 1 h using a semi-dry transfer machine (Amersham). After 1 h blocking with PBS, 0.1% Tween-20 (Promega) and 5% milk membranes were incubated at 4°C with monoclonal mouse ANTI-FLAG M2 antibody (1:10,000 dilution, Sigma-Aldrich). After washes with PBS-0.1% Tween, membranes were incubated at room temperature for 1 h with the secondary antibody goat anti-mouse IgG HRP conjugate (1:4000 dilution, Millipore). Detection of samples was achieved by incubating membranes with SuperSignal West Pico Chemiluminescent Substrate (Thermo Scientific).

### Immunofluorescence

T-REx293-cFXN cells grown on glass coverslips were treated with 100 nM MitoTracker Red (Life Technologies) for 30 min, fixed with 4% paraformaldehyde in DMEM at 37°C for 15 min and permeabilized for 5 min at room temperature with 0.3% Triton X-100 in PBS containing 5% FBS. Samples were then washed and blocked with 5% FBS in PBS and incubated with monoclonal anti-FLAG M2 (Sigma-Aldrich) (1:2000 dilution) at 4°C overnight. The following day, slides were washed and incubated with the secondary antibody AlexaFluor-488 goat anti-mouse antibody (Life Technologies) (1:1000 dilution) for 60 min at room temperature. After three washes, slides were mounted with ProLong Gold Antifade Reagent that includes 4′,6-diamidino-2-phenylindole (DAPI) nuclear stain and imaged using Delta vision Inverted microscope (Olympus).

### Development of TALENs and CRISPRs

TALEN-binding domains were designed and developed following published guidelines (Cermak et al., 2011). Each binding domain was created by two rounds of the ‘Golden Gate’ assembly method (Cermak et al., 2011), where each TAL module was linked together and cloned on a single plasmid backbone using type IIS restriction enzymes *Bsa*I and *Esp*3I. Full-length constructs were subsequently cloned on either pcGoldy-TALEN (TALEN-I3.1, -I3.2, I3.3 and I4) or on pCS2TAL3-RRR/DDD (TALEN-I2) expression vectors for expression in human cells. The Golden Gate TALEN kit and expression vectors were obtained from Addgene (Cambridge, MA, USA).

CRISPRs were designed following the Church's protocol (Mali et al., 2013). The expression vectors for the gRNAs were generated by ordering a 455 bp DNA fragment (composed of U6 promoter, target sequence, guide RNA scaffold and termination signal) as gBlocks from IDT (Leuven, Belgium) and then cloning them in pUC19 plasmid. The hCas9 expression vector was instead obtained from Addgene.

### Restriction digestion and surveyor (Cel-I) assays

TALEN-I2, -I3.1 and -I3.2 target sites harbour a unique restriction site that can be used to identify TALEN-induced insertions/deletions (indels). Briefly: 2×10^5^ T-REx293-cFXN cells were plated in 6-well plate and transfected 48 h after using Lipofectamine 2000 with 2.5 μg of vectors encoding the relative TALENs. Genomic DNA was extracted 48 h after transfection using the Wizard genomic DNA purification kit (Promega). Q5 Hot Start High-Fidelity DNA Polymerase (NEB) was used to amplify the relative TALEN target regions (Table 2).

**Table 2.**
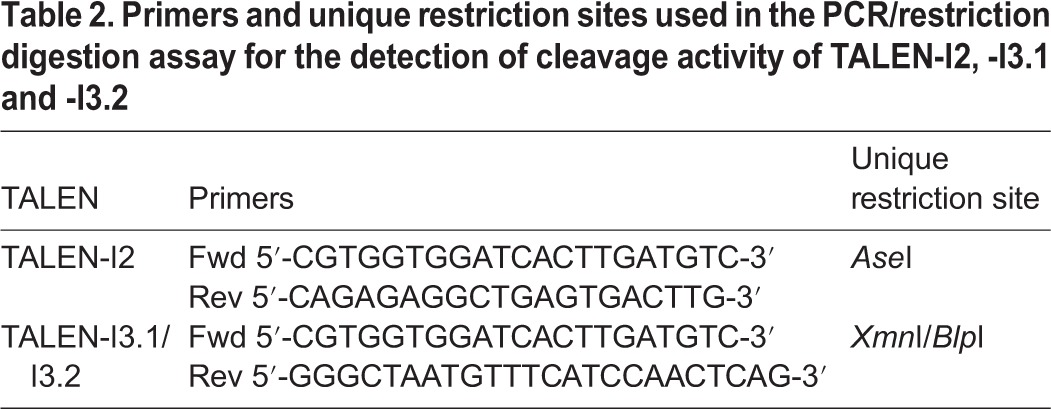
**Primers and unique restriction sites used in the PCR/restriction digestion assay for the detection of cleavage activity of TALEN-I2, -I3.1 and -I3.2**

PCR products were purified using ZymoClean kit (ZymoResearch) and digested with the respective unique restriction enzyme (Table 2). Digestion products were then analysed either on 2% agarose or precast 10% TBE polyacrylamide (Life Technologies) gels, depending on the size of the original PCR product. The presence of TALEN-induced indels was confirmed by identification of uncut products.

TALEN-I3.3-, -I4- and CRISPRs-induced indels were analysed using the Surveyor kit assay (Transgenomic). Briefly, 2×10^5^ T-REx293-cFXN cells were plated in 6-well plates and transfected for 48 h using Lipofectamine 2000 with 2.5 μg of vectors encoding the relative TALENs or with 2.5 μg of vectors encoding Cas9 nuclease and relative gRNA guides. Genomic DNA was extracted 48 h after transfection using Quick-gDNA Microprep (ZymoResearch). Optimase high fidelity polymerase (Transgenomic) was used to amplify the relative TALEN target regions (Table 3). The generated PCR products were directly treated with the commercially available Surveyor kit (Transgenomic) in accordance with the manufacturer's instructions. Results were visualized on a precast 10% TBE polyacrylamide gel (Life Technologies).

**Table 3.**
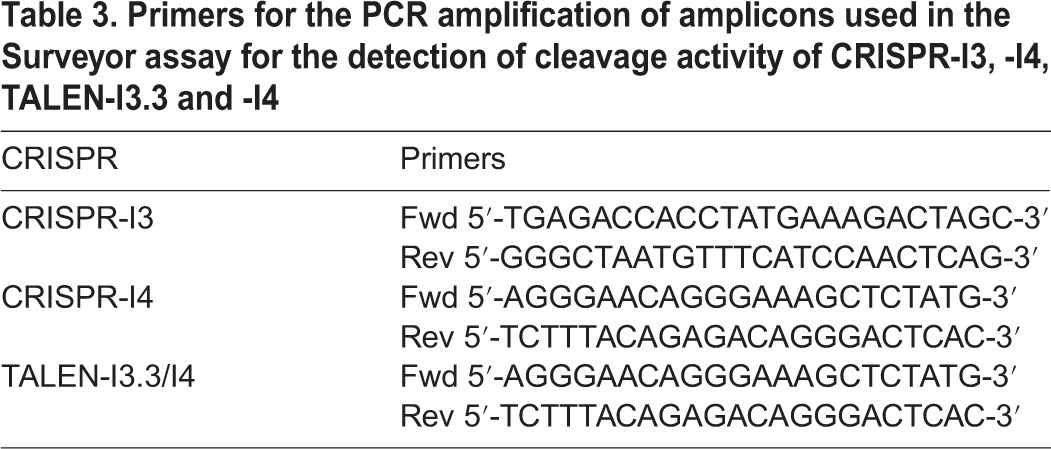
**Primers for the PCR amplification of amplicons used in the Surveyor assay for the detection of cleavage activity of CRISPR-I3, -I4, TALEN-I3.3 and -I4**

### Gene targeting

T-REx293-cFXN cells were transfected, following the protocol described above, with 0.5 μg of pFSVpur-LoxP-TC-I4 targeting construct, 1 μg of CRISPR-I4 vector and 1 μg of hCas9 vector or with 1.5 μg of pFSVpur-LoxP-TC-I4, 0.5 μg of CRISPR-I4 and 0.5 μg of hCas9. As a negative control, cells were transfected with 1.5 μg of pFSVpur-LoxP-TC-I4 and 1 μg of hCas9. Transfected cells were expanded for 48 h. Selection with 0.4 μg/ml puromycin was started and carried on for 15 days. Twenty puromycin resistant colonies for each transfection were isolated and expanded. Genomic DNA for PCR screening was isolated for each one of the selected colonies using Quick-gDNA Microprep (ZymoResearch). The target and construct specific primers used for PCR screening were 5′-TGTAGTCCCAGCTCTCTGGAGG-3′ and 5′-GCATTCTAGTTGTGGTTTGTCC-3′, respectively. PCR screening was carried out using KAPA HiFi hotstart DNA polymerase (Kapa Biosystems).
